# The prevalence of physical inactivity in Iranian adolescents and the impact of economic and social inequalities on it: results of a National Study in 2018

**DOI:** 10.1186/s12889-020-09618-0

**Published:** 2020-10-02

**Authors:** Ghobad Moradi, Farideh Mostafavi, Bakhtiar Piroozi, Bushra Zareie, Marzieh Mahboobi, Mohammad Aziz Rasouli

**Affiliations:** 1 Social Determinants of Health Research Center, Research Institute for Health Development, Kurdistan University of Medical Sciences, Pasdaran Ave, Sanandaj, Iran; 2 Clinical Research Development Center, Kowsar Hospital, Kurdistan University of Medical Sciences, Sanandaj, Iran; 3 Center for Communicable Disease Control, Ministry of Health and Medical Education, Tehran, Iran

**Keywords:** Socioeconomic status, Inequalities, Physical inactivity, Concentration index, Adolescent, Iran

## Abstract

**Background:**

This study aims to determine the prevalence of physical inactivity in Iranian adolescents aged 10–12 years and the impact of socioeconomic inequality on it.

**Methods:**

In this descriptive study, the study population consisted of 10–12 years old adolescents from an Iranian population from Kurdistan, Fars and Markazi provinces in 2018. The sample size was 1590 individuals. The sampling method was cluster sampling. Data was collected using demographic questionnaire, modifiable activity questionnaire (MAQ) and socioeconomic questionnaire. Cut points on the MAQ for light activity, moderate activity and heavy activity were MET< 3, MET = 3–6 and MET> 6, respectively. Linear and logistic regression were used to estimate the final model and the Oaxaca analysis method was applied. All analyses were performed in Stata/SE 14.0.

**Results:**

Of the 1590 participants, 52.82% were male. The results showed that 25.79% of the subjects were physically inactive and 7.30% engaged in moderate physical activity during the week. The average physical activity during 1 week was more in boys than in girls (*P*-value< 0.05). Adolescents of mothers with secondary and high school education were more likely to have physical inactivity than mothers with a high school diploma or higher (AOR: 1.35, 95% CI: 1.02–1.77). The concentration index was −.11, indicating a greater concentration of physical inactivity in adolescents with low socioeconomic levels.

**Conclusion:**

One-fourth of the study population had physical inactivity in this age group. Socioeconomic levels, parental literacy, and sex of adolescents were associated with the level of physical activity.

## Background

Physical inactivity (PI) is a public health concern that is considered a potential risk factor for adverse health outcomes worldwide. The World Health Organization (WHO) reports the prevalence of PI to be more than 80% in adults and 23% in adolescents. This rate varies considerably across countries. Reports indicate that the prevalence of PI is higher in the eastern Mediterranean region, the US, Europe and the Western Pacific region than in other parts of the world [1]. Urban and industrial life, advances in technology, economic development and globalization have led to rapid changes in lifestyle and PI in individuals, leading to an increase in the prevalence of related chronic diseases such as coronary heart disease, colon cancer, hypertension, stroke, breast cancer, type 2 diabetes and osteoporosis [2].

Childhood is a duration of fast physical and cognitive development [3]. Studies show that many behavioral risk factors for chronic diseases, including PI, are rooted in childhood and that the patterns of behavior developed during this period remain relatively stable in adolescence and adulthood [4, 5]. PI leads to obesity, reducing working memory and brain health in children [6]. Therefore, it seems that children are an important target group for interventional and preventive activities. Any intervention in this group can be very effective in controlling the chronic diseases epidemic in adulthood [3].

Today’s adolescents are less active than their peers decades ago. Watching television and computer games are known to be the main causes of PI in adolescents. There are also other factors in Iran, such as living in small houses and apartments, the phenomenon of single adolescent or the low number of adolescent in each family, lack of appropriate sports facilities in the community and schools, pressure of lesson plans for high school students, preference of education than other aspects of life, disregard for sports at school and home and lack of security in the community that affect inactivity and lower activity of children and adolescents [3, 7].

One of the most important health indicators that influence one’s attitude, behavior, and exposure to risk factors is socioeconomic status (SES). There is evidence to suggest that the lower the SES of a person, the worse their health status. There is, in fact, a social gradient in health that moves from the top to the bottom of the socioeconomic spectrum and creates gaps in health outcomes such as infection rates, mortality and disability across different social classes worldwide [8]. In the 2014 study by Finger et al., the physical activity and SES of parents of German children aged 11–17 years were examined. The results showed that higher education of parents was negatively correlated with the level of PI of children [9]. Rey-López’s study [10] had similar results; its results showed an inverse relationship between socioeconomic factors such as parental education and child PI. A systematic review was also conducted to investigate socioeconomic factors affecting physical activity by Ragna Stalsberg et al. The results indicated a positive relationship between socio-economic factors and physical activity, especially the impact on leisure time [11].

To our knowledge, there is little information available on the status of physical activity in adolescents in Iran. So far, very few studies have been conducted to investigate the effect of socio-economic inequalities on PI in Iranian adolescents. Therefore, the aim of the present study was to determine the prevalence of PI in Iranian adolescents aged 10–12 years and the effect of socioeconomic factors and economic inequalities on it.

## Methods

### Study setting and participants

The population of this cross-sectional study was 10–12 years old children in fourth, fifth and sixth grades of elementary school. The final sample size was 1590 individuals. The multi-stage sampling method involving systematic classification, clustering and random methods were used. For the first step, the provinces of Iran were divided into three geographical clusters of 9, 9 and 13 provinces. One province was randomly selected from each cluster including Kurdistan province in the west, Markazi in the center and Fars in the east. In the next step, one city was randomly selected in the selected provinces including Marivan in Kurdistan, Saveh in Markazi and Garash in Fars. The number of children in the age range were 13,513 (Saveh), 9864 (Marivan) and 2561 (Garash). In order to reduce the error, the sample size of each cluster was selected proportional to the size of that cluster. Six elementary schools (three for girls and three for boys) were randomly selected from the primary schools in each city and 526 samples (263 female students and 263 male students) were randomly selected from each of the 10 to 12-year-old students (Grade 4, 5 and 6). Prior to the study, written consent was obtained from all parents and trained public health experts were used to collect data. Questionnaires used in this study included demographic information questionnaire, modifiable activity questionnaire (MAQ) [12] and socioeconomic status questionnaire which were completed by adolescents and parents at home based on the protocol.

In the MAQ questionnaire, each physical activity was weighted according to its metabolic equivalent (MET). One MET was the amount of energy consumed by a resting person every minute. Then, the level of physical activity was calculated in terms of hours of activity per week (MET-h/WK) and the subjects were divided into three groups based on their overall physical activity: light activity (MET< 3), moderate activity (MET = 3–6) and heavy activity (MET> 6). Based on the studies, the reliability (Interclass correlation coefficient was calculated to assess the reliability) of this questionnaire is 0.97 and its validity is 0.47 (Pearson correlation coefficients was calculated to assess the validity) [12]. To determine SES, questions were asked about parental assets. The SES of each province was measured based on an assets index computed for each province using the data on assets ownership of households (percent of the households that own computers, washing machines, dishwashers, vacuum cleaners, refrigerators, freezers, fridge freezers, car and Internet. Using a principal component analysis (PCA) method, an asset index was calculated and SES has been determined by the assets index of the households computed (1 = poorest, 5 = richest) based on the asset index.

### Statistical analysis

To determine the SES, we used the method proposed by O’Donnell and Moradi, et al. [13, 14]. Accordingly, a socioeconomic status questionnaire consisting of a number of assets (including vacuum cleaners, computers or laptop, refrigerators or separate refrigerators, washing machine, Gas cooler, LCD or LED TV, mobile phone, dishwasher, furniture, oven, microwave oven, internet access, personal car, landline telephone, personal home and number of rooms) was used. PCA first identified the variables that had the most impact on the variance of all variables and then a new SES variable was constructed based on these variables. The PCA gives each asset a specific coefficient or weight then extracts a linear combination of the variables with the most variance. It then eliminates this variance and finds the second linear combination that describes the largest proportion of the residual variance, and continues this procedure. In this study, we developed an asset index using the PCA method. Five quintiles were constructed using the mean of this variable which divided the statistical population into 5 groups: very poor, poor, middle, rich and very rich. Finally, the PI variable was compared between the two very poor and very rich groups.

Logistic regression was used to evaluate the relationship between prevalence of outcome variables in different socioeconomic subgroups. The highest socioeconomic group was considered as the baseline. The model began with the baseline model and then added just significant determinants and explained the effect of each determinants contributing to inequality.

Concentration index (CI) and concentration curve (CC) methods were used to measure inequality. The CI quantitatively shows the degree of inequality at the income distribution level of a health variable. To calculate the relative CI, individuals are first sorted by socioeconomic status, and then the cumulative percentage of the population is plotted against a cumulative percentage of the health variable (enjoyment rate) to create the CC. CI values vary from + 1 to − 1 [14]. It is one of the most common indices in calculating income inequality and socioeconomic status. Negative values indicate that the PI variable is concentrated among people with poor socioeconomic status and the CC is above the equality line. But positive values indicate that the PI variable is concentrated among the rich, and when the distribution of health among all individuals is the same, the CI will be zero. The Kakwani method was used to calculate the CI based on the following formula:

1 $$ C=\frac{2}{\mu}\;\mathrm{COV}\;\left({y}_{i,}\;{R}_i\right) $$

In this formula, C is the concentration index, Cov is covariance, yi is health variable, Ri is economic rank, and μ is mean health variable. Due to the limitations of this index for binary data, CI was normalized based on the method used by Wagstaff according to the following formula in which C is the standard concentration index and μ is mean health variable [15].

2 $$ \mathrm{Wc}=\mathrm{C}/\left(1-\mu \right) $$

After measuring the inequalities, the decomposition concentration index was used to determine the contribution of each of the determinants to inequality. According to the Wagstaff method, we considered a linear regression model in relation with PI variable (y) and a set of determinants (*x*_*k*_):

3 $$ {y}_i=\alpha +{\sum}_k{\beta}_k{x}_{ki}+{\varepsilon}_i $$

i is the average i^th^ person, *β*_*k*_ is the regression coefficient, *ε*_*i*_ is error coefficient or interpersonal changes. Based on the relationship between *y*_*i*_ and *x*_*k*_ in formula 3, we write C for PI as follows:

4 $$ C=\sum \left(\frac{\beta_k{\overline{x}}_k}{\mu}\right){C}_k+\frac{GC_{\varepsilon }}{\mu } $$

In this formula, inequality generally consists of two explained and unexplained parts. In the explained part, μ is the mean health variable, *β*_*k*_ is the regression coefficients for PI on the available determinants, ${\overline{x}}_k$ is the mean of the determinants or *x*_*k*_ , *C*_*k*_ is the concentration index C for the *x*_*k*_ determinant and in the unexplained part, $\frac{GC_{\varepsilon }}{\mu }$ is the residual error in the total C for *ε*_*i*_ [14].

In this study, PI was considered as the response variable and Chi-square test was used to estimate the prevalence of response variable at each level of demographic variables. Multivariate logistic regression was used to estimate the final model based on variables with *p* <  0.1 in chi-square test and calculation of univariate OR and AOR. All analyses were performed in Stata/SE 14.0.

## Results

Of the 1590 participants, 410 (25.79%) were physically inactive, 116 (7.30%) had moderate physical activity and 1064 (66.92%) had heavy physical activity during the week.

Among the participants, 696 (44%) cycling, 633 (40%) football or handball, 613 (38%) went running, 485 (30%) volleyball, 347 (22%) swimming, 226 (14%) hiking or outings, 212 (13%) skating, 199 (12%) did martial arts, 133 (8%) ping pong or badminton, 124 (7%) dance or rhythmic movements, 116 (7%) gymnastics and endurance sports, 100 (6%) basketball, 78 (5%) wrestling, (2%) bodybuilding or weightlifting, 8 (0.05%) skiing, and 90 people (5%) did other physical activities.

The mean (standard deviation) of total physical activity in terms of (MET-h/WK) was 40.72 (76.2). The mean (SD) of physical activity among girls was 30.87 (58.44) and among boys was 49.61 (88.28) MET hours per week. The mean (SD) of physical activity among fourth-graders was 40.1 (77.9), fifth graders 38.7 (64.7) and sixth graders 43.5 (88.3). Table 1 shows physical activity in terms of sex, educational grade, BMI, maternal education, father education, age of father, age of mother, highest education level of both parents, and household size. Mean physical activity during 1 week was significantly different between boys and girls (*P* <  0.05).

**Table 1:** Physical inactivity in terms of demographic variables

Variable^a^	Subgroups	Number (%)	Physical Activity (MET)		*P*-value
			≤ 6	> 6	
Sex	Man	836 (52.58)	241 (28.83)	595 (71.17)	< 0.001
	Female	754 (47.42)	285 (37.80)	469 (62.20)	
Age groups	10	224 (14.09)	67 (29.91)	157 (70.09)	0.290
	11	768 (48.30)	268 (34.90)	500 (65.10)	
	12	597 (37.55)	191 (31.99)	406 (68.01)	
Household size	4≤	927 (58.30)	295 (31.82)	632 (68.18)	0.525
	> 4	593 (37.30)	198 (33.39)	395 (66.61)	
BMI	Under 5 percentiles	102 (6.42)	37 (36.27)	65 (63.73)	0.732
	5–50 Percentiles	484 (30.44)	163 (33.68)	321 (66.32)	
	50–85 Percentiles	561 (35.28)	176 (31.37)	385 (68.63)	
	Overweight & Obese	429 (26.98)	143 (33.33)	286 (66.67)	
Parental’ level of education	Non-academic	1241 (78.05)	413 (33.28)	828 (66.72)	0.560
	Academic	320 (20.13)	101 (31.56)	219 (68.44)	
Mother education	Illiterate and primary	591 (37.17)	201 (34.01)	390 (65.99)	0.074
	Mid school & high school	397 (24.97)	145 (36.52)	252 (63.48)	
	Diploma & academic	565 (35.53)	168 (29.73)	397 (70.27)	
Father education	Illiterate and primary	390 (24.53)	130 (33.33)	260 (66.67)	0.300
	Mid school & high school	414 (26.04)	146 (35.27)	268 (64.73)	
	Diploma & academic	735 (46.23)	227 (30.88)	508 (69.12)	
Mother age	< 35	507 (31.89)	165 (32.54)	342 (67.46)	0.761
	35–44	759 (47.74)	244 (32.15)	515 (67.85)	
	> 45	166 (10.44)	49 (29.52)	117 (70.48)	
Father age	< 35	111 (6.98)	37 (33.33)	74 (66.67)	0.566
	35–44	916 (57.61)	294 (32.10)	622 (67.90)	
	> 45	421 (26.48)	124 (29.45)	297 (70.55)	
Total	–	1590 (100)	526 (33.08)	1064 (66.92)	–

^a^Number of missing values for each variable (%):

Age group 1(0.06); Household size 70 (4.40); BMI 14 (0.88); parental’ level of education: 29 (1.82); Mother education: 37 (2.33); father education: 51(3.21) Mother age:158 (9.94); Father age: 142 (8.93);

In Table 2, univariate OR is presented for those variables in Table 1 that had a significant relationship at *P* = 0.2 with physical activity level, using univariate and multivariate logistic regression. The OR for the variables of sex and maternal education had a significant relationship with physical activity level (*P* <  0.05). In multivariate logistic regression, sex and maternal education at secondary and high school level had a significant relationship with physical activity level (*P* <  0.05).

**Table 2:** Univariate and multivariate logistic regression of physical inactivity

Variable		Number (%)	OR (95%CI)	*P*-value	AOR (95%CI)	*P*-value
**Sex**	Male	836 (52.58)	Ref.	**–**	Ref.	–
	Female	754 (47.42)	1.50 (1.22–1.85)	< 0.001	1.56 (1.26_1.93)	< 0.001
**Mother education**	Diploma & academic	565 (36.38)	Ref.	–	Ref.	–
	Illiterate and primary	591 (38.06)	1.22 (0.95_1.56)	0.119	1.17 (0.91_1.51)	0.209
	Mid school & high school	397 (25.56)	1.36 (1.04_1.79)	0.027	1.35 (1.02_1.77)	0.033

Effects of two variables sex and Mother education, were adjusted in multivariable model

**Fig. 1:**
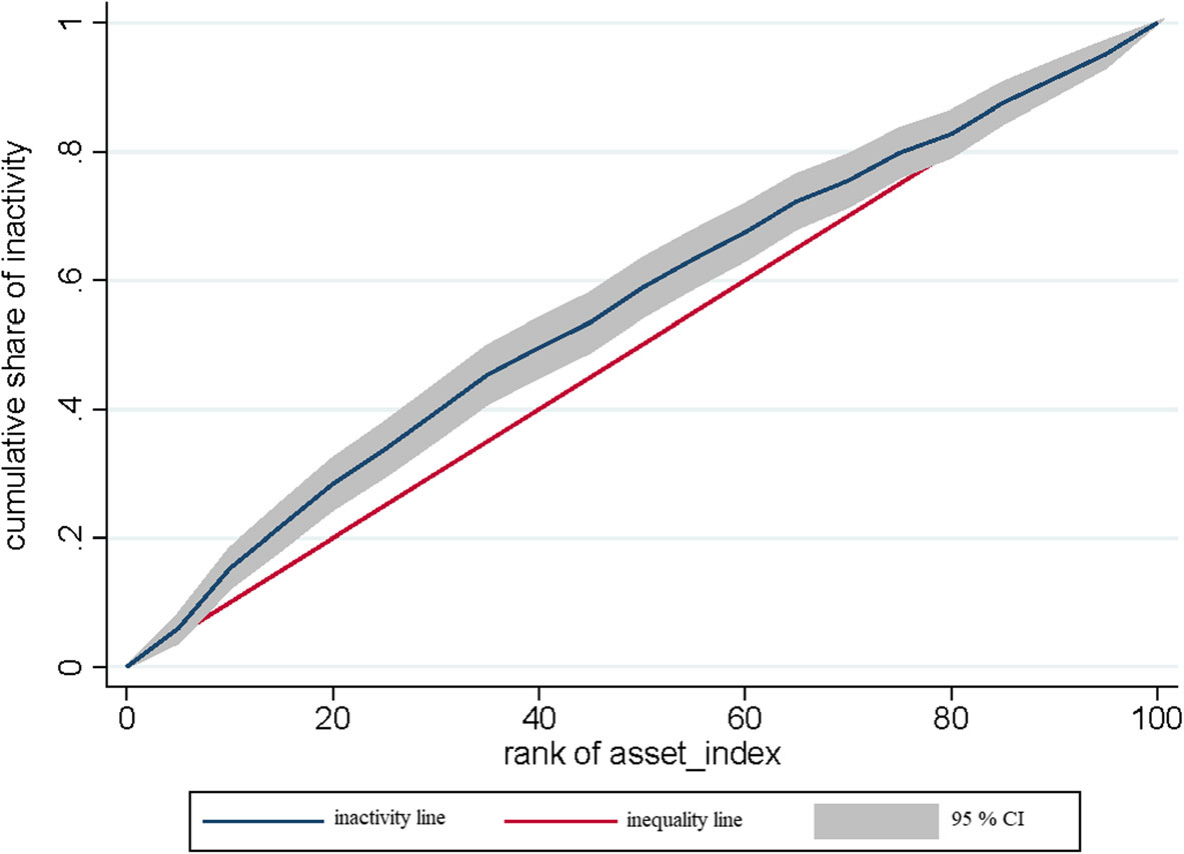
The concentration curve for physical inactivity in adolescent in Iran

Also, the concentration index for PI was negative (CI = − 0.11), indicating inadequate activity in adolescents with low socioeconomic status. The Fig. 1 indicated pro-poor inequality for PI, CC of PI in is above the equality line, indicating a greater concentration of PI in the poorer group of the community.

Table 3 shows the results of CI decomposition for PI in adolescents. The reference group of 10-year-old male students with parents under 35 years of age and high school diploma or academic education was considered the highest economic class and household size of less than 4 children. Unfavorable economic situation (− 537.41%), father’s education (385.87%) and household size (123.11%) respectively had the greatest role in creating inequalities in PI. The contribution of mother’s education in creating inequality was − 181.17. The contribution of parents’ age in creating inequality at age 35 to 44 was 45.39 and 8.96, respectively, and the contribution of age was − 52.85 (Table 3).

**Table 3:** Decomposition of concentration results for physical inactivity

Variables	Coef	Elast	CI	Cont to C	C%
Sex					
Male					
Female	−0.011	−0.084	0.049	−0.004	−15.92
Age					
10					
11	−0.041	−0.195	−0.004	0.000	3.27
12	0.042	0.183	− 0.076	−0.013	−52.85
Economic statue					
Poorest SES	0.073	0.191	− 0.741	− 0.142	−537.41
2th SES	0.031	0.086	− 0.362	−0.031	− 118.02
Middle SES	0.053	0.140	0.015	0.002	8.02
4th SES	− 0.009	−0.025	0.380	−0.009	−37.25
5th SES					
Size of family					
4≤					
> 4	−0.058	−0.256	−0.126	0.032	123.11
Mother Education					
Uneducated & Elementary	−0.057	−0.386	0.123	−0.047	−181.17
Middle & High school	0.007	0.028	0.047	0.001	5.03
Diploma and academic					
Father Education					
Uneducated & Elementary	0.103	0.859	0.118	0.102	385.87
Middle & High school	0.062	0.186	−0.037	−0.007	−26.64
Diploma and academic					
Mother age					
< 35					
35–44	−0.007	−0.050	−0.046	0.002	8.96
> 45	0.026	0.057	−0.010	−0.000	−2.17
Father age					
< 35					
35–44	0.050	0.399	0.030	0.012	45.39
> 45	0.004	0.021	−0.052	−0.001	−4.19

Coeff Marginal effects, Elast elasticity, CI Concentration index of the social determinants, Cont to C Contribution to the overall concentration index, C% unadjusted percentage calculated on the overall explained portion of the C

## Discussion

Based on the findings of this study, about 26% of adolescents were physically inactive. Female adolescents and adolescents with less educated mothers were significantly more physically inactive. Also, according to the centralized index, inequality was seen in the PI rate, so that the PI was concentrated in lower socioeconomic classes. Adverse economic conditions, paternal education, and household size were the most important factors associated with PI inequality.

PI is one of the most important risk factors for mortality worldwide. It is one of the pests of industrial life that is increasing every year due to advances in science and technology [16].

The results showed that about one quarter of adolescent in the study had PI. The results of other studies conducted in Iran and other parts of the world also show a high prevalence of PI in adolescent all over the world [17–19]. The results of the study by Kelishadi et al. on 6–18 years children showed that 24% of them were physically inactive and the average physical activity in boys was higher than in girls, which is consistent with the present study [19]. A systematic review to assess PI in Arab countries also found that the prevalence of PI was higher in girls than in boys [20]. Differences in physical activity can be influenced by gender-defined roles or societal norms and values, which may result in women having less access to resources for exercise and have fewer choices for leisure time [21, 22]. Also, differences in psychological and behavioral aspects can be effective in making these differences [23].

The findings showed that adolescents with mothers with low levels of literacy had a higher chance of PI than others. Mothers with more education appear to be more aware of the importance of regular physical activity in adolescents [24, 25]. However, the results of some studies are different and show that the level of physical activity in adolescent decreases as mothers’ education and employment levels increase [26]. These differences may be justified by different cultural and family backgrounds. Providing proper awareness and education about the necessity of physical activity in adolescents is one of the critical issues that should be considered for families. One message of this study to improve physical activity is to increase mothers’ education level.

Another determinant of health and well-being in all periods of life is socio-economic status, which is considered an important factor in creating health inequalities in populations [27]. In this study, the factors affecting inequality were investigated using the decomposition model. The findings showed that low economic status, level of education of the father and the family size were the most important factors in creating inequality. CI analysis showed that people with low economic status had less physical activity than other adolescent. These adolescent may not have access to sports facilities and equipment due to their cost constraints or may be reluctant to do so due to lack of time and lack of access to safe and appropriate sports facilities [28]. A study by Prins et al. on Dutch adolescents showed that adolescents’ lack of involvement in sport activities was highly correlated with lack of access to sports venues, parks and green spaces, and the socioeconomic status of households in a neighborhood [29]. On the other hand, some scholars believe that people in the upper classes are most aware of the goals of a regular physical activity promotion program [30, 31]. However, some studies have had different results and have shown that children in the upper classes may have less physical activity for reasons such as having a more prosperous life, more consumption of fast foods, more use of vehicles, computer games, mobile phones, etc. [32, 33].

In the present study, fathers’ education level also played an important role in creating inequalities: children of fathers with low educational level were more inactive. This difference may be due to the low level of literacy, awareness and attitude of the head of household that who is mostly the father towards the benefits of physical activity of adolescent and not providing suitable conditions for physical activity in children according to their life preferences. It seems that when education level of parents increases, they have more information and they can better encourage their adolescents to do physical activity. Similar results were obtained in the Eduardo Gonzalo-Almorox study, which examined the effect of socio-economic inequalities on leisure-time inactivity of Spanish adolescents. The results of this study showed that the education level of household head and family income were the main socio-economic inequalities affecting child inactivity [34]. Also, in the present study, household size or the number of individuals in the family was one of the factors affecting the inactivity of adolescent. Adolescents with more than 4 family members had more PI than families with less than 4 members.

In a study carried out in Kurdistan in 2014, the factors affecting inequality in PI of adolescent aged 10–12 were investigated using Oxaca model. The results of the study showed that the PI of individuals in the lower socioeconomic classes was higher than the others. In the initial model, increased maternal literacy, improved socioeconomic status, and improved living place were all effective in increasing physical activity. Environmental problems such as lack of open space, parks, or sports fields often make it difficult that some adolescent engage in sufficient physical activity.

It seems that, given the contradictions in the results of various studies, education on the importance of public health and adopting healthy behaviors during childhood and adulthood due to the dangers of inadequate physical activity are essential to have a healthy community away from economic and social differences. School-based interventions, with the involvement of students’ families, and multidisciplinary interventions such as active commuting from/to school, active Physical training lessons, active school break, sleep health promotion can ultimately lead to increased physical activity in children and adolescents [35, 36].

In the present study, cycling and sports such as soccer or handball were more common than other physical activities, and skiing accounted for the lowest percentage of physical activity in adolescents. This difference, on the one hand, may be due to the availability of sports facilities for both sexes, the costs and seasonality of a sport, and, on the other hand, due to parents’ views on its impact on adolescents future. These results indicate that boys and girls have different expectations and interests in choosing their physical activities, which is very important for policy-making and planning.

One of the limitations of this study may be recall bias due to questions about subjects’ activities in a past period, which were attempted to be reduced as much as possible by training and protocol preparation. Another limitation is uncertainty about the measure of association in a cross-sectional study. However, the present study with a large sample size provides valuable evidence on family and behavioral risk factors affecting PI in Iranian adolescents, which can justify further related studies, effective interventions, and planning and policy making.

## Conclusion

One quarter of the study population had PI, which is alarming for this age group. The situation of the girls was worse. PI was more concentrated in adolescents with poorer socioeconomic status. Socioeconomic levels, parental literacy, and children’s sex were factors affecting the level of physical activity. Improving the socioeconomic status of households, increasing the education of parents and especially mothers, and paying more attention to groups with lower socioeconomic status are recommended to improve physical activity in adolescent. Large-scale national policies are recommended to promote physical activity in adolescents. Increase in parental knowledge through local mass media (TV, Radio) or other communication strategies may be associated with more support for participation in physical activity.

Domestic governments have a key role to play in promoting physical activity. Design and construction of safe places like parks, soccer fields, and walking trails are associated with more outdoor play especially in adolescents with lower economic levels that needs national policies.

## Supplementary information

**Additional file 1.**

## Data Availability

The datasets used and/or analyzed during the current study can be made available by the corresponding author on reasonable request.

## Abbreviations

PI: Physical inactivity
MAQ: Modifiable activity questionnaire
SES: Socioeconomic status
PCA: Principal component analysis
SD: Standard deviation
