# Supplementary material for: The prevalence of physical inactivity in Iranian adolescents and the impact of economic and social inequalities on it: results of a National Study in 2018
Source: BMC Public Health. 2020 Oct 2;20:1499. doi: 10.1186/s12889-020-09618-0 (PMC7532599; doi:10.1186/s12889-020-09618-0)
Supplement: Supplementary file 1 — Additional file 1. [file 12889_2020_9618_MOESM1_ESM.docx]

Physical activity asset

Questionnaire

| **Instruction:**  **Dear Parents, this questionnaire is designed to assess your child's physical activity. Please answer the following questions carefully.**  **Kind Regards,**  ***Social Determinants of Health Research Center, Kurdistan University of Medical Sciences.*** |
| --- |

1. **How many times does your child done at least 20 minutes of strenuous exercise in the last 14 days that increase his or her breathing and heart rate. (Strenuous exercise such as basketball, jogging or fast cycling). Please consider the time attending a sports class as well.**

(Please select one option only)

|  |
| --- |

1. Not at all

|  |
| --- |

1. Once to twice

|  |
| --- |

1. Three to five times

|  |
| --- |

1. Six to eight times

|  |
| --- |

1. Nine or more
2. **How many times does your child done at least 20 minutes of light exercise in the last 14 days on average, that does not increase his or her breathing and heart rate. (Light exercise such as light exercise, walking or slow cycling. Please also consider the time attending a sports class as well.**

(Please select one option only)

|  |
| --- |

1. Not at all

|  |
| --- |

1. Once to twice

|  |
| --- |

1. Three to five times

|  |
| --- |

1. Six to eight times

|  |
| --- |

1. Nine or more
2. **How many hours on average does your child spend watching TV, playing computer or video games per day?**

(Please select one option only)

|  |
| --- |

1. Not at all

|  |
| --- |

1. An hour or less

|  |
| --- |

1. Two to three hrs

|  |
| --- |

1. Four to five hrs

|  |
| --- |

1. Six hrs or more
2. **How many hours on average does your child spend using a tablet or mobile phone per day?**

(Please select one option only)

|  |
| --- |

1. Not at all

|  |
| --- |

1. An hour or less

|  |
| --- |

1. Two to three hrs

|  |
| --- |

1. Four to five hrs

|  |
| --- |

1. Six hrs or more
2. **Has your child been a member of an in-school or out-of-school sports team in the past year? If yes, how many times has she or he participated in team or individual sports or competitive sports?**

(Please select one option only)

|  |
| --- |

1. Not at all

|  |
| --- |

1. Once

|  |
| --- |

1. Twice

# Please mention any other activities that your child has been involved in?

1. …………..
2. …………..
3. …………..
4. …………..

Record of physical and recreational activities over the past year

# What type of activities has your child done more than 10 times in his / her free time during the last year. (Don not consider the time that has been spent in school sports classes)

# Running

# Martial arts

# Gymnastics/Endurance sports

# *Soccer/* *Handball*

# Basketball

# Volleyball

# Cycling

# Ping pong/ Badminton

# Fitness/ Weightlifting

# Swimming/ Diving

# Wrestling

# Skiing

# Skating

# Mountaineering/ Out town sightseeing

# Rhythm physics (Dancing)/ Aerobics

# Others (please mention)

| **Please write each of the activities you mentioned above in the table below, mark the months your child was physically active and note the approximate time of the activity as well.** | | | | | | | | | | | | | | |
| --- | --- | --- | --- | --- | --- | --- | --- | --- | --- | --- | --- | --- | --- | --- |
| Type of activity | January | February | March | April | May | June | July | August | September | October | November | December | Number of times per week | Number of minutes per week |
|  |  |  |  |  |  |  |  |  |  |  |  |  |  |  |
|  |  |  |  |  |  |  |  |  |  |  |  |  |  |  |
|  |  |  |  |  |  |  |  |  |  |  |  |  |  |  |
|  |  |  |  |  |  |  |  |  |  |  |  |  |  |  |
|  |  |  |  |  |  |  |  |  |  |  |  |  |  |  |
|  |  |  |  |  |  |  |  |  |  |  |  |  |  |  |
|  |  |  |  |  |  |  |  |  |  |  |  |  |  |  |
|  |  |  |  |  |  |  |  |  |  |  |  |  |  |  |
|  |  |  |  |  |  |  |  |  |  |  |  |  |  |  |
|  |  |  |  |  |  |  |  |  |  |  |  |  |  |  |

**Dear Parents, please answer the following questions:**

**Child’s Mother info:**

# What is your date of birth?

Please state in DD/MM/YYYY e.g. 17/08/1960

/ /

# Which of the below best describes the highest level of education you have completed?

Primary (left before 16, before finishing secondary school)

Secondary (left at 16 or ‘O’ Levels or GCSE’s)

Further Education (after 16 or ‘A’ Levels/BTEC)

Undergraduate degree or equivalent

Postgraduate Degree or equivalent

Professional Qualification/PhD

Other

Prefer not to say

None of the above

**Child’s Father info:**

# What is your date of birth?

Please state in DD/MM/YYYY e.g. 17/08/1960

/ /

# Which of the below best describes the highest level of education you have completed?

Primary (left before 16, before finishing secondary school)

Secondary (left at 16 or ‘O’ Levels or GCSE’s)

Further Education (after 16 or ‘A’ Levels/BTEC)

Undergraduate degree or equivalent

Postgraduate Degree or equivalent

Professional Qualification/PhD

Other

Prefer not to say

None of the above

| What is your child date of birth? Please state in DD/MM/YYYY e.g. 17/08/1960  / /  **How many members has your family got?**  ........... |
| --- |

**How many times does your child brush their teeth per day?** ...........

**How many times does your child use Fluoride per day?**  ...........

| 1 | Are your family members covered by health insurance?  Yes  No | 10 | Do you have a washing machine at home?  Yes  No |
| --- | --- | --- | --- |
| 2 | Are your family members covered by supplemental health insurance?  Yes  No | 11 | Do you have an ovenat home?  Yes  No |
| 3 | Do you have vacuum cleaner at home?  Yes  No | 12 | Do you have a microwave at home?  Yes  No |
| 4 | Do you have Laptop or computer at home?  Yes  No | 13 | Do you have Internet access at home?  Yes  No |
| 5 | Do you have an air conditioner or fan coil at home?  Yes  No | 14 | Do you have a personal car?  Yes  No |
| 6 | Do you have refrigerator at home?  Yes  No | 15 | Does the house you live in belong to you?  Yes  No |
| 7 | Do you have LED/LCD television at home?  Yes  No | 16 | How many bedrooms does your house have?  Yes  No |
| 8 | Do you have any furniture at home?  Yes  No | 17 | ______ |
| 9 | Do you have a landline at home??  Yes  No |  | ______ |
